# Waste Material Classification Based on a Wavelength-Sensitive Ge-on-Si Photodetector

**DOI:** 10.3390/s24216970

**Published:** 2024-10-30

**Authors:** Anju Manakkakudy Kumaran, Andrea De Iacovo, Andrea Ballabio, Jacopo Frigerio, Giovanni Isella, Lorenzo Colace

**Affiliations:** 1 Department of Industrial, Electronic and Mechanical Engineering, Roma Tre University, Via Vito Volterra 62, 00146 Rome, Italy; anju.manakkakudykumaran@uniroma3.it (A.M.K.); lorenzo.colace@uniroma3.it (L.C.); 2 Eye4NIR S.r.l., Via Bartolomeo Colleoni, 14, 23801 Calolziocorte, Italy; andrea.ballabio@eye4nir.com; 3 LNESS Dipartimento di Fisica, Politecnico di Milano, Via Anzani 42, 22100 Como, Italy; jacopo.frigerio@polimi.it (J.F.); giovanni.isella@polimi.it (G.I.)

**Keywords:** dual-band photodetector, material classification, machine learning algorithm, optical sensor

## Abstract

Waste material classification is critical for efficient recycling and waste management. This study proposes a novel, low-cost material classification system based on a single, voltage-tunable Ge-on-Si photodetector operating across the visible and short-wave infrared (SWIR) spectral regions. Thanks to its tunability, the sensor is able to extract spectral information, and the system effectively distinguishes between seven different materials, including plastics, aluminum, glass, and paper. The system operates with a broadband illuminator, and material identification is obtained through the processing of the photocurrent signal at different bias voltages with classification algorithms. Here, we demonstrate the basic system functionality and near real-time classification of different waste materials.

## 1. Introduction

The escalating volumes of non-degradable waste materials like plastics, glass, paper, and aluminum pose significant challenges to modern societies, which require increasingly novel approaches to waste management. While eliminating these materials entirely may be unrealistic, innovative approaches to waste management are essential. Fortunately, advancements in automated sorting techniques offer a promising solution for improving recycling efficiency. These techniques utilize advanced sensors and mechanical methods to swiftly sort materials based on their characteristics. A computer system determines their destination, and mechanical processes separate them accordingly. Despite recent advances, both in terms of costs and functionalities, further optimization of automated sorting systems is required to stimulate their adoption, both in industrial recycling facilities and for low-volume waste management facilities closer to the waste generation point.

Automated waste sorting employs both direct and indirect techniques. Direct sorting methods use material properties like magnetism, conductivity, and density, employing techniques such as magnetic and gravity separation for simultaneous characterization and sorting [1]. Indirect sorting methods first analyze material properties using sensors, followed by a separate physical process to achieve material separation. Techniques such as X-ray fluorescence (XRF), laser-induced breakdown spectroscopy (LIBS), and optical sensor-based sorting fall within this category [2]. Optical sensor-based sorting detects material properties through light interactions like reflectance and absorption, often using machine learning to quickly and accurately classify materials by their optical signatures [3,4]. While optical sensors offer a powerful tool for waste sorting, other sensor technologies have their own niches and limitations. For instance, X-ray sensors might be crucial for identifying hazardous materials within the waste stream and eddy, and magnetic sensors may excel at identifying specific material types, but they often have limitations regarding the size of objects they can effectively detect. Also, the effectiveness of LIBS is constrained by factors such as the temperature of plasma excitation, the collection efficiency of emitted light, and the intensity of the observed spectral lines. Additionally, a key limitation of LIBS is the extremely small sample volume, which can lead to sampling bias [5].

Optical sensors offer manyfold advantages in waste management; they provide non-contact sensing, fast and non-destructive analysis, and support for multi-criteria sorting based on color, shape, size, and texture. The introduction of spectral imaging, such as multi/hyperspectral imaging, combining spatial and spectral data, is expected to increase the classification capability significantly, thanks to the exploitation of the material optical properties [6]. Moreover, machine learning algorithms act as intelligent assistants, analyzing large datasets to identify patterns and make automated decisions, enhancing automated sorting processes for the faster, more consistent completion of repetitive tasks [7]. These technologies find applications not only in waste management [8,9,10,11] but also in fields like food, pharmaceuticals, forensics, and surface analysis [12,13,14,15].

Unfortunately, the widespread adoption of hyperspectral systems faces challenges such as the high cost and the need for fast computers, sensitive detectors, extensive training data, and complex algorithms to accurately classify materials based on visual appearance [16]. An easier approach consists of ditching imaging capabilities while focusing on the spectral analysis of waste materials. While the initial cost of implementing spectroscopy can be a factor, compact spectrometers offer significant advantages. These instruments provide faster analysis and achieve higher spectral resolutions with respect to imaging systems [17]. Therefore, they are useful in more complex waste streams, where material differentiation based on chemical composition is crucial.

A material’s unique characteristics are often revealed across multiple spectral domains, and several systems realized with off-the-shelf electronics and operating in the SWIR and MIR have already been demonstrated for high-accuracy and cost-effective waste classification [18,19,20,21,22,23]. Despite the outstanding results reported in the literature, spectroscopic systems often rely on several different light sources or on diffractive optical elements, thus implying high complexity and increased costs.

To address these disadvantages, we propose a material classification system based on a voltage-tunable photodetector, which is based on a germanium-on-silicon epitaxial structure with two back-to-back connected photodiodes. Operating at a low bias (−1 V < Vb < 1 V), it can selectively detect either within the 400–1100 nm or 1000–1600 nm windows. In addition, by sweeping the applied voltage, its spectral response can be continuously changed, providing information related to the spectral characteristics of the optical radiation, thus allowing for the analysis of the reflectance characteristics of waste materials without resorting to bulky spectral analysis equipment.

Here, we demonstrate the exploitation of this device for the characterization and classification of waste materials through automatic data analysis with machine learning algorithms. This approach offers both in-depth analysis and rapid identification of diverse materials, including four types of polymers, glass, aluminum, and paper. By combining the power of spectral analysis and machine learning, the proposed system may represent a significant advancement in waste sorting technology, paving the way for accurate, efficient, and non-destructive material classification, and fostering research and development efforts in this promising field.

## 2. Materials and Methods

### 2.1. Ge-on-Si Dual-Band Photodetector

**Figure 1:**
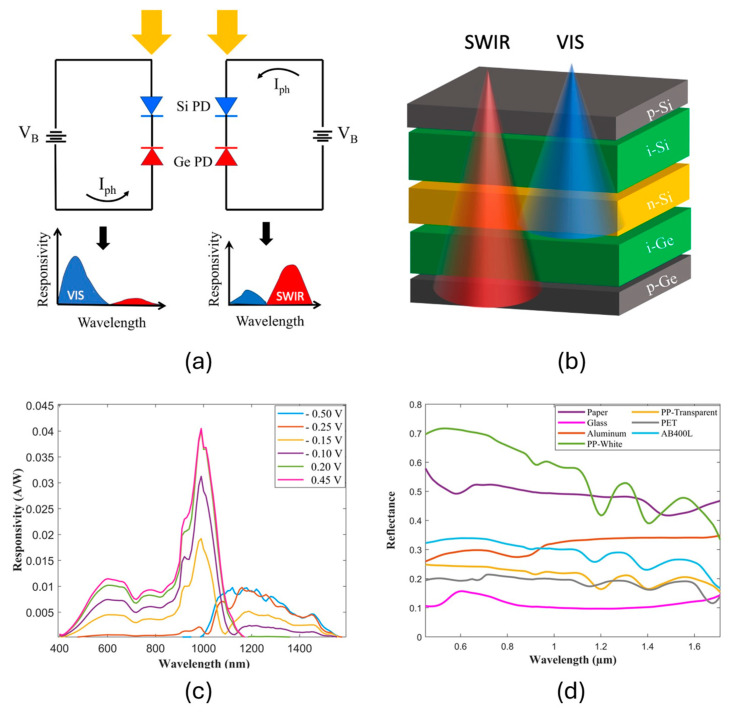
(**a**) Schematic representation of device and its principle of operation. (**b**) Epitaxial structure. (**c**) Spectral responsivity of dual-band photodetector at different voltage biases. (**d**) Reflectance spectra of different waste materials.

The key innovation behind this system lies in the electrically tunable dual-band photodetector [24]. This patented device is provided by Eye4NIR S.r.l. (Calolziocorte, Italy), and it is fabricated through monolithic integration, essentially combining two back-to-back connected photodiodes (PDs)—each featuring a unique absorption band—into a single unit [as illustrated in Figure 1a]. By applying a specific voltage bias (Vb), we can electronically control which photodiode is active. Figure 1b shows the epitaxial stack structure of the device.

A positive bias (Vb) enhances the sensitivity of the Si photodiode to shorter, visible wavelengths (400–1100 nm), as shown by the upshift in the spectral response. Conversely, reversing the bias polarity activates the second photodiode (Ge), enabling the detection of longer, near-infrared wavelengths (SWIR: 1000–1600 nm). This is evident in Figure 1c, where the measured spectral response progressively decreases in the visible range and increases in the SWIR range as the voltage bias is swept from +0.45 V to −0.5 V. This makes the device suitable for extracting spectral information about the impinging optical radiation. Figure 1d shows the typical reflectance spectra of several different waste materials gathered with a compact spectrometer system from OceanOptics (Orlando, FL, USA). All the materials show peculiar characteristics in the 400–1600 nm wavelength range, thus suggesting that the double-diode device herein described could be effectively employed to obtain spectral information from the light reflected by the waste samples.

### 2.2. Sample Set

**Figure 2:**
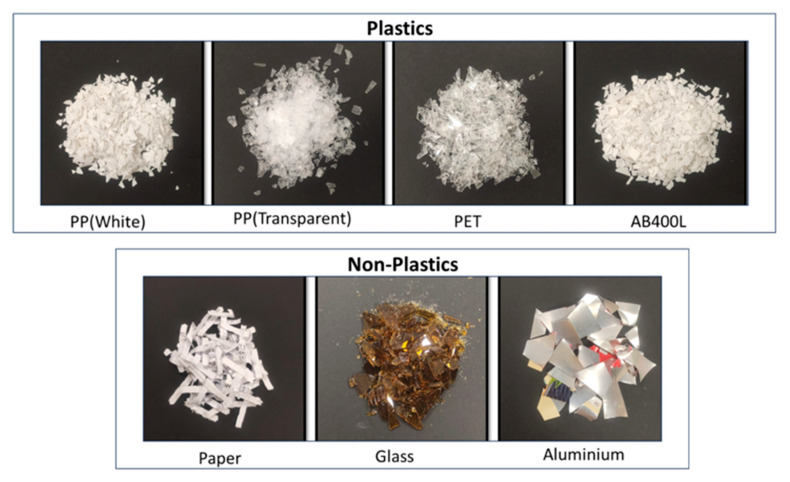
Samples used in the research.

This study focuses on classifying waste materials by selecting commonly encountered waste items: paper, glass, aluminum, and four types of plastic, namely polyethylene terephthalate (PET), polypropylene (PP) in both transparent and white forms, and AB400L, a composite polymer of polylactic acid (PLA) and polybutylene succinate. Due to the limitations of SWIR spectroscopy for analyzing dark-colored samples [25], only white and transparent materials were used in this study. It is important to acknowledge that the materials are in the form of flakes and pellets, which may not fully represent the physical properties of real-world waste. Notably, except for the plastics, all materials were sourced from a waste bin. Photographs of the samples used in the research are given in Figure 2.

### 2.3. Measurement System

**Figure 3:**
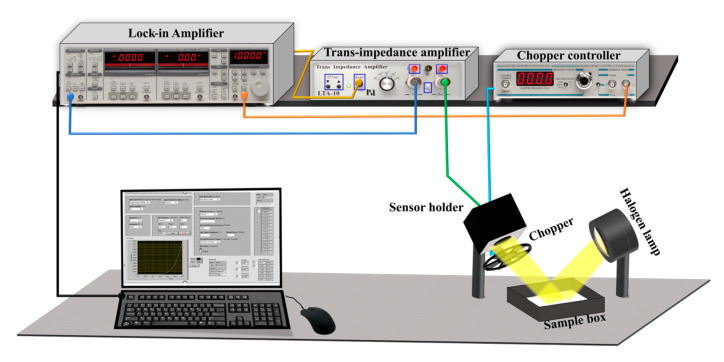
Schematic representation of experimental setup.

A sketch of the experimental measurement setup is shown in Figure 3. The Ge-on-Si photodetector is housed in a 3D-printed plastic holder (the “sensor head”), limiting the field of view of the sensor in order to reduce the effects of unwanted light reflections. Illumination is provided by a halogen lamp. The diffused reflected light from the sample is modulated by a chopper mounted in front of the aperture of the sensor head. The transimpedance amplifier (TIA) amplifies and converts the photocurrent generated by the photodetector into a proportional voltage signal with gain and provides the bias voltage. A lock-in amplifier is used to enhance the sensitivity, improving the signal-to-noise ratio and canceling the photodetector dark current, thanks to the homodyne detection operated by the lock-in at the chopper frequency. A PC running a custom LabVIEW program manages the measurement providing the data acquisition and timing. Preliminary tests allowed for the optimization of the system geometry (i.e., distance and angle between the sensor, the sample, and the light source).

Preliminary measurements explored a voltage bias range of −1 V to 1 V with two different sampling intervals: 20 mV and 40 mV. Both approaches yielded similar accuracy. Notably, within this range, we observed significant variations in the spectral response of the sensor, particularly between −0.5 V and −0.2 V, owing to differences in the reflectance spectra of the different material samples. Such spectral fingerprints allowed for effective differentiation between materials. Based on these findings, we have narrowed the focus of further measurements to the −0.5 V to −0.2 V range with a sampling resolution of 6 mV.

### 2.4. Data Acquisition

The data acquisition parameters were optimized to guarantee stable measurements. All measurements were taken using a time integration of 100 ms and a time delay of 1 s. To ensure accurate measurements and minimize potential artifacts from preferential reflection angles within the material grains, each sample box underwent shaking prior to every measurement. Over the course of six separate days, fifty measurements were collected for each of the seven materials, resulting in a robust dataset comprising 6 distinct day-based datasets of 350 photocurrent curves each. This comprehensive approach yielded a total of 2100 curves across all materials, providing a solid foundation for the subsequent analysis and discussion of the results.

The timing parameters used for data acquisition imply long measurement times. While this is acceptable during the system training, faster acquisitions are desirable during operation. In order to optimize the performance of the system, we investigated the classification accuracy for data acquired with a shorter delay and integration time (Section 3.2).

Before classification, all the photocurrent curves underwent normalization within the 0–1 interval using a min–max normalization method. Normalization is a crucial preprocessing step that transforms all variables to a common scale. This ensures that each variable contributes equally to the subsequent analysis, regardless of its original unit or range.

**Figure 4:**
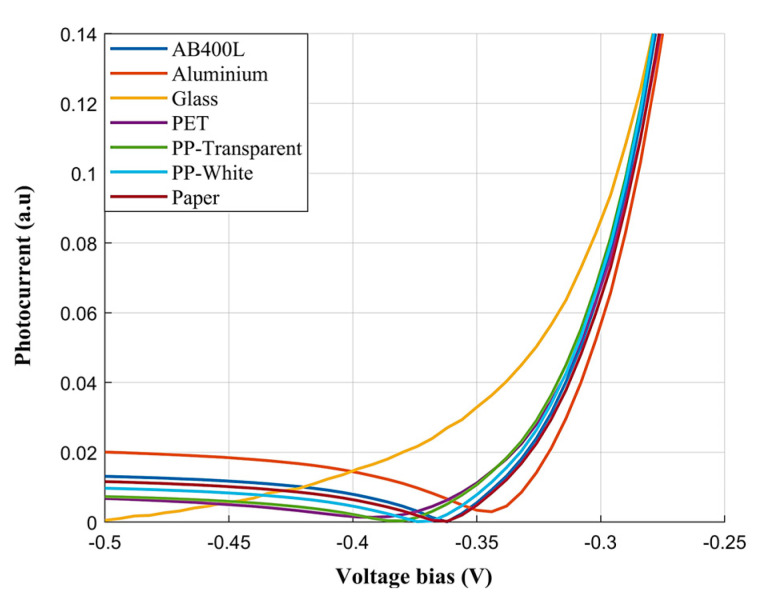
Average photocurrent spectra for each material as a function of voltage bias.

Figure 4 shows the photocurrent response of various materials obtained through the tunable dual-band photodetector, following data normalization and zooming in on a small magnitude range for better visibility. The curves for glass and aluminum are easily distinguishable from the other materials under investigation. Glass features a low photocurrent across the whole voltage range, with a sharp increase at −0.4 V, indicating low light reflection until this threshold. Aluminum, on the other hand, features a much higher photocurrent, especially at lower voltages, due to its high reflectivity. PET and PP transparent have similar photocurrent curves, showing consistently low photocurrents, which indicates their high transparency. Materials like AB400L, PP white, and paper exhibit unique photocurrent characteristics, especially between −0.5 V and −0.35 V, allowing for easy identification. In general, while the photocurrent–voltage characteristics may appear similar, they exhibit several peculiarities, such as the zero-crossing voltage and the photocurrent ratios at different voltages, which can be effectively exploited by classification algorithms.

## 3. Results and Discussion

### 3.1. Multivariate Analysis

The classification process involved an analysis of the six individual datasets separately. This approach allows for the assessment of the performance of the model on each day-based dataset. Additionally, classification was performed on the combined dataset encompassing all six days. This step helps in evaluating the impact of factors like measurement day, temperature, device performance, or external lighting conditions, which can introduce intra-class variations within the same material type. By comparing the classification accuracy of the combined data with the day-based datasets, we can gain a more comprehensive understanding of the robustness and reliability of the classification model. During the model training, a 5-fold cross-validation approach was implemented to guarantee robust model evaluation and mitigate overfitting [26].

Based on a 5-fold cross-validation, the dataset of 350 spectra was divided into five equal subsets of 70 spectra each. In each iteration, four folds (280 spectra) were used for training, while one fold (70 spectra) served as the testing set, resulting in an 80% training and 20% testing distribution. This approach maximized dataset utilization and provided a comprehensive evaluation of model performance, with each fold serving as the testing set once across the five iterations. To further enhance the analysis, principal component analysis (PCA) was employed for efficient dimensionality reduction [27]. PCA reduces the number of features while preserving key information, which is particularly beneficial for high-dimensional datasets and helps mitigate overfitting by minimizing the influence of irrelevant features. The first three principal components obtained from PCA were used to build the classification model. The same approach was applied to the combined dataset of 2100 spectra, divided into five subsets of 420 spectra, with four folds (1680 spectra) for training and one fold (420 spectra) for testing.

To ensure optimal classification performance for the diverse material types under investigation, a comprehensive evaluation of various machine learning algorithms was undertaken. This evaluation encompassed established algorithms such as the support vector machine (SVM), k-nearest neighbors (KNN), linear discriminant analysis (LDA), and random forest (RF) [28]. By exploring these diverse approaches, we aimed to identify the algorithm that best leverages the unique characteristics of our dataset, ultimately achieving the highest classification accuracy. The classifier’s ability to learn and accurately classify the data is measured by classification accuracy, which is defined as the percentage of correctly predicted samples out of the total samples.

Our evaluation of various machine learning algorithms revealed the k-nearest neighbors (KNN) algorithm as the leading performer. Although all methods exhibited good accuracy (detailed in Table 1), KNN consistently achieved high accuracy across all day-based datasets and was the best performer for combined data, effectively classifying the diverse materials under investigation. This superior performance across datasets strengthens the selection of KNN as the primary classification algorithm for this application.

**Figure 5:**
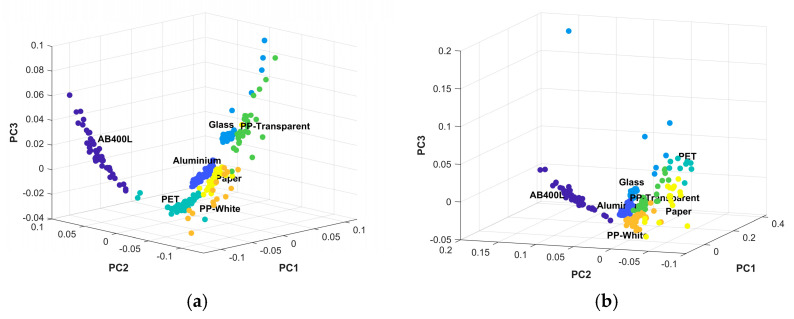

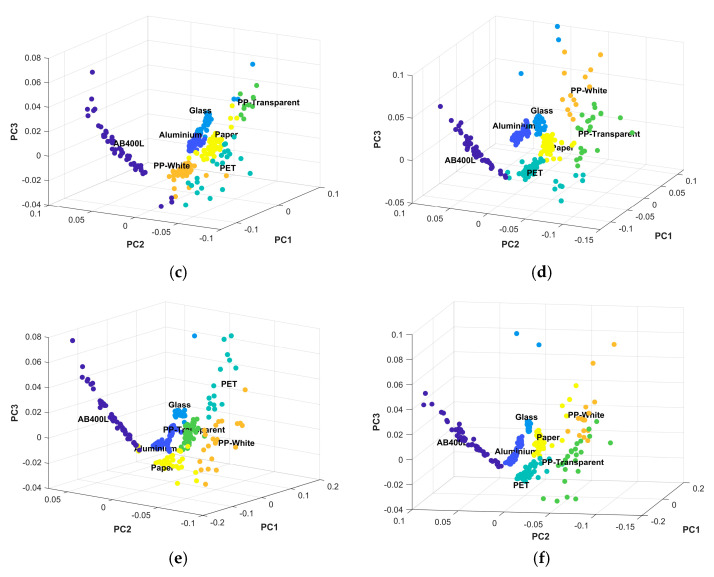
Clustering results of the classification model based on the first three principal components across a six-day dataset, with subfigures (**a**) showing day 1, (**b**) day 2, (**c**) day 3, (**d**) day 4, (**e**) day 5, and (**f**) day 6. Different colors identify different materials.

Figure 5 illustrates the clustering results of the KNN-based classification model, which is based on the first three principal components that together account for 95% of the variance in the dataset. The analysis of the six dataset PC clusters indicates that materials such as AB400L and glass are well separated, suggesting that PCA effectively captures the differences among these materials and can distinguish them from others. However, some overlap is observed primarily among PP white and transparent, which are closely clustered due to their similar spectral characteristics, as they are made from the same base material. Additionally, instances where PET clusters closely with paper, PP transparent, and aluminum further illustrate the challenges in classification. Despite this overlap, the figures clearly show that all materials are distinctly separated and can be easily classified, demonstrating that PCA components are valuable for classifying materials effectively.

**Figure 6:**
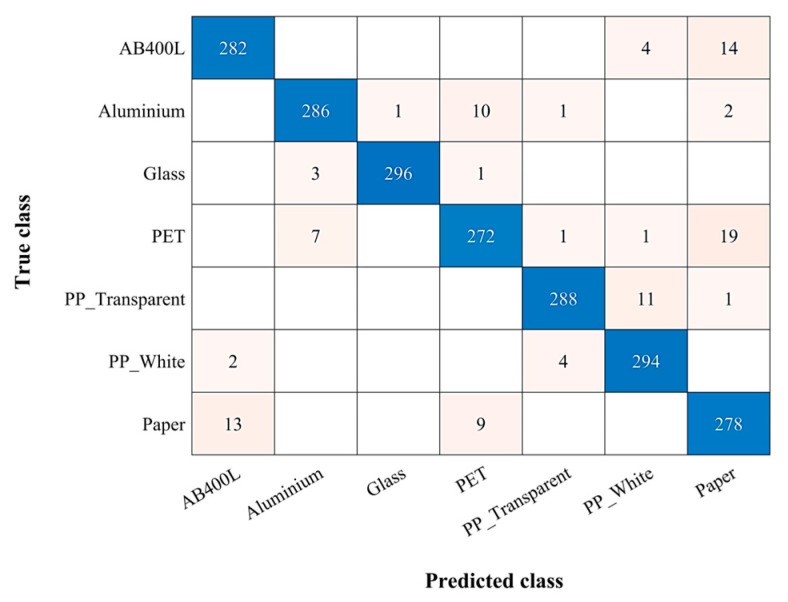
Confusion matrix of combined data for KNN classifier.

Figure 6 shows the confusion matrix [29] for the combined dataset, revealing a 95% classification accuracy achieved by the KNN method. A crucial parameter in KNN is the number of neighbors (k) considered for classification. In this study, we opted for k = 1. This choice emphasizes localized patterns within the parameter space, which is particularly beneficial for achieving high accuracy. Additionally, Euclidean distance [30], a common metric known for its simplicity and effectiveness, was used to measure the proximity between data points. The chosen parameters also help in minimizing overfitting and contribute to accurate predictions on unseen data. Notably, these same parameters were consistently applied throughout all individual dataset analyses, ensuring methodological uniformity across the entire study.

The KNN classifier demonstrated good performance across all materials, with all classifications exceeding 90% accuracy (based on true positive rate (TPR) and false negative rate (FNR) metrics [31]). Some materials achieved even better results: glass with 98.7% and PP white and transparent reached 98% and 96% accuracy, respectively. While the overall performance was outstanding, a closer look reveals some materials with significant misclassification rates. AB400L had a 6% misclassification rate, being primarily confused with paper and PP white. PET presented the biggest challenge, experiencing a 9.3% misclassification rate, with paper and aluminum being the most frequent misclassifications. Paper exhibited a 7.3% misclassification rate.

### 3.2. Optimization of Data Acquisition Parameters for Rapid Measurements

The system was trained (combined data) using data acquired with a 100 ms integration time and a 1 s time delay (i.e., the time the system waits between the application of the bias voltage and the acquisition of the measurement). This methodology was chosen to ensure stable and repeatable measurements, which are crucial for building a reliable training dataset. The 100 ms integration time allows for sufficient averaging of the signal, reducing noise and improving the accuracy of the measurement. The 1 s time delay, on the other hand, provides a buffer period between measurements, ensuring that transient effects do not affect the stability of the recorded data. However, this configuration only allows for slow measurements (1.1 s for each data point, plus data transmission latency), and it appears incompatible with the fast classification needed by industrial recycling facilities. For this reason, we investigated the change in classification performance for data acquired with a shorter delay and integration time. It should be noted that data employed for the training of the classification model were not changed, and only new datasets were acquired with reduced measurement times.

Two distinct configuration settings were employed: Configuration 1 utilized a 500 ms delay and a 30 ms integration time, while Configuration 2 used a 100 ms delay while retaining the same 30 ms integration time. The selection of a 30 ms integration time for both configurations was a deliberate choice. While lower integration times can further enhance speed, they often come at the expense of a reduced signal-to-noise ratio (SNR), potentially impacting classification accuracy. In our specific application, a 30 ms integration time provided an acceptable trade-off between speed and accuracy. Maintaining the same integration time allows us to isolate the effect of varying the delay time on classification accuracy without introducing additional factors related to the measurement SNR.

**Figure 7:**
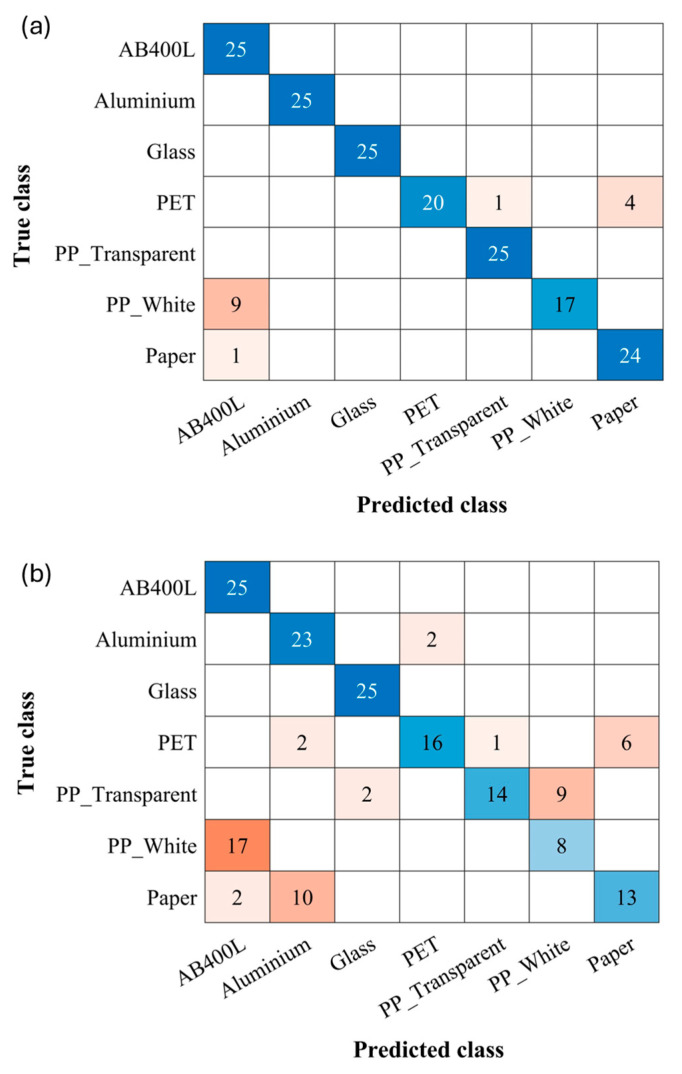
(**a**) Confusion matrix of classification model with 500 ms time delay. (**b**) Confusion matrix of classification model with 100 ms time delay.

Figure 7a,b show the confusion matrices for the classification model with time delays of 500 ms and 100 ms, respectively, both using a 30 ms integration time. A 500 ms delay yielded a promising overall accuracy of 91.48% [Figure 7a], with occasional misclassifications observed for specific materials, namely PET, PP white, and paper. However, reducing the time delay to 100 ms [Figure 7b] significantly compromised the system performance, resulting in a substantial drop in the overall accuracy to 70.86%. This shorter delay also led to a marked increase in misclassifications, particularly for PP materials, PET, and paper. Interestingly, the classification accuracy for AB400L and glass is not affected by the reduction in delay time, showing that the spectral reflectivity characteristics of such materials are very well identified with respect to the other samples.

**Figure 8:**
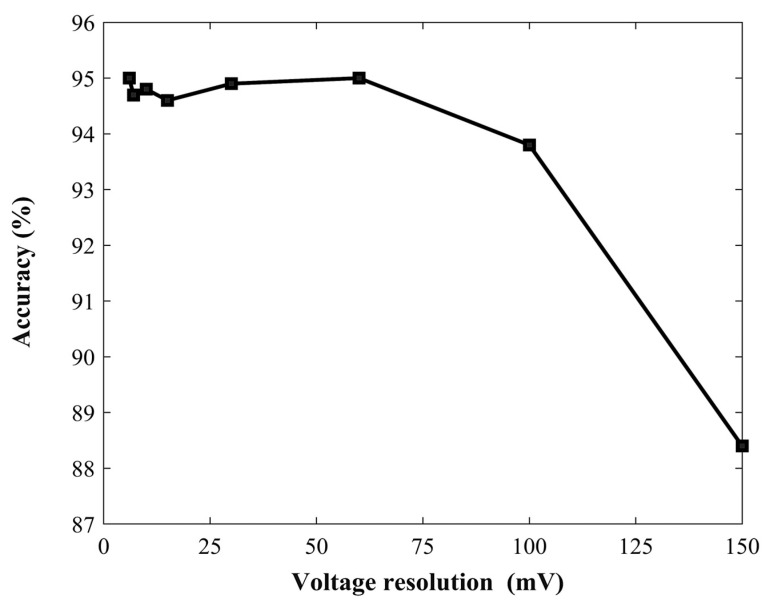
Impact of voltage resolution on the overall accuracy of the classification model.

Even though good accuracy has been retained for the 500 ms delay time, measurements are still too slow for practical applications, and further optimization is needed. A feasible approach would consist of the employment of multiple voltage-tunable photodetectors, each biased at a specific voltage, operating in parallel, thus allowing for the simultaneous acquisition of multiple data points. To this extent, and for the sake of the simplicity and economy of the system, a small number of voltages should be employed. In order to evaluate the impact of reduced voltage resolution on the performance, we decreased the number of voltage steps while maintaining a uniform spacing across the voltage range. Figure 8 and Table 2 explore the relationship between the voltage resolution and classification accuracy for the combined dataset. Table 2 shows the overall accuracies achieved with various voltage resolutions and corresponding voltage steps. Notably, the results demonstrate that classification accuracy remains high and stable (>95%) for resolutions up to 60 mV (5 voltage steps). However, a significant drop in accuracy is observed at larger voltage steps (100 mV and 150 mV). This trend is further emphasized by the visual representation in Figure 8.

In real-life scenarios, it is not always useful to discriminate and classify many different materials, but a rough sorting of materials in wider classes can be accepted for the sake of simplicity. For this reason, we explored the performance of the system by reducing the number of classes and considering three different frameworks: (i) plastic vs. non-plastic materials, (ii) plastics, glass, aluminum, and paper, and (iii) only plastic materials to be classified based on the specific polymer.

**Figure 9:**
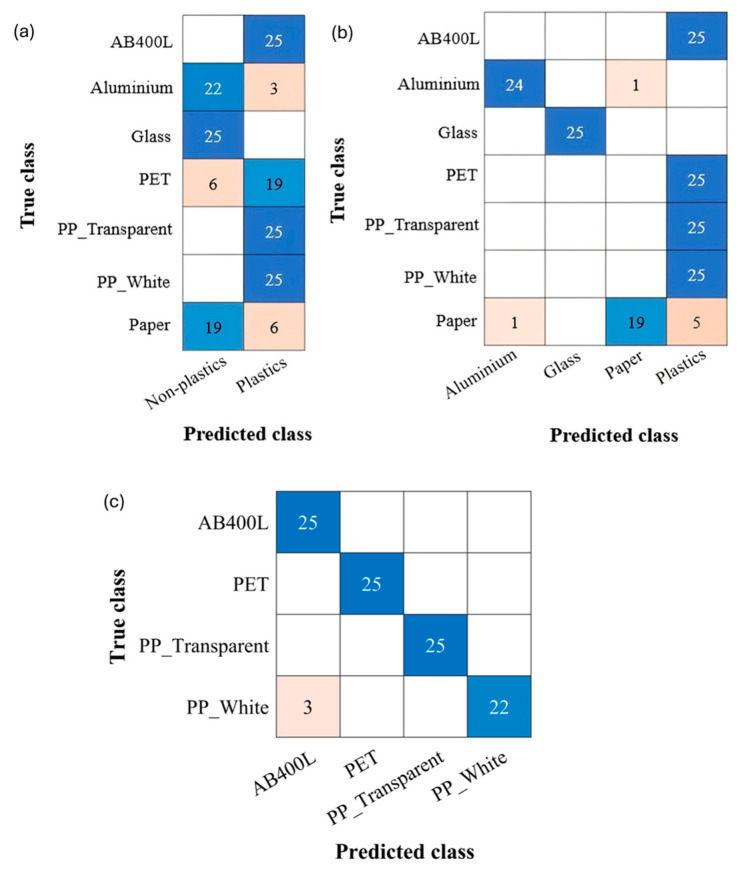
(**a**) Confusion matrix for model 1 with 500 ms time delay. (**b**) Confusion matrix for model 2 with 500 ms time delay. (**c**) Confusion matrix for model 3 with 500 ms time delay.

Figure 9 shows the confusion matrices for the validation of the three classification models. In all cases, the models were trained with the original datasets, and tests were executed by collecting 25 new photocurrent curves for each material with a 500 ms delay time and 30 ms integration time.

When distinguishing only between plastics and non-plastics, the system yielded promising results, showing a 91.42% accuracy [Figure 9a]. Differentiation between plastics, glass, aluminum, and paper achieved an accuracy of 94.85% [Figure 9b]. This is noteworthy because all plastic materials were consistently classified correctly.

Focusing only on plastics yielded improved accuracy, with an impressive 97% achieved [Figure 9c]. All materials except white PP were correctly identified.

## 4. Conclusions

In this work, we demonstrated the feasibility of a cost-effective, laboratory-based material classification system. Utilizing a single, bias-tunable Ge-on-Si dual-band photodetector combined with classification algorithms, the system successfully differentiated between seven distinct materials. This accomplishment paves the way for further development towards more sophisticated and adaptable material identification solutions. We also investigated the possibility of obtaining faster classification by reducing the integration and delay time of the measurement system, demonstrating that good accuracy can still be maintained even when halving the overall measurement time, with respect to the one employed for the acquisition of training data. Considering the acquisition of all 50 data points/curves at a 100 ms delay and 30 ms integration time, a single measurement requires 6.5 s. This allows for the recognition of static objects only. However, we discussed how further improvements can be obtained by reducing the amount of data points (i.e., increasing the voltage step), exploiting a multisensor array of photodetectors, and simplifying the classification system with a reduced set of output classes.

Finally, it should be mentioned that all experiments were conducted in a laboratory environment on controlled samples, and thus the results cannot be immediately transferred into industrial, high-TRL systems. Nevertheless, we present our study as a proof of concept aiming at the demonstration of the voltage-tunable photodetector for waste classification.

Further investigation will be required to understand the performance of the system in the presence of contaminants and moisture or mixed waste materials. Anyhow, we believe the proposed system could be best suited for user-level applications (like smart garbage bins), rather than for large waste management plants.

## Figures and Tables

**Table 1:** Classification accuracy comparison across four different methods over considered dataset (best results are highlighted in bold).

Dataset	KNN	LDA	RF	SVM
**1st set**	**98.6%**	94.3%	97.4%	97.7%
**2nd set**	**94.9%**	88.9%	93.7%	93.1%
**3rd set**	**95.1%**	91.1%	**95.1%**	94.9%
**4th set**	97.7%	94.6%	**98.0%**	**98.0%**
**5th set**	**96.6%**	92.0%	94.9%	96.3%
**6th set**	97.4%	95.1%	**97.7%**	97.1%
**Combined data**	**95.0%**	86.8%	94.1%	94.0%

**Table 2:** Overall classification accuracy at different voltage resolution levels.

Voltage Interval/Steps	Voltage Resolution (mV)	Overall Accuracy (%)
50	6	95.0
40	7	94.7
30	10	94.8
20	15	94.6
10	30	94.9
5	60	95.0
3	100	93.8
2	150	88.4

## Data Availability

Data underlying the results presented in this paper are not publicly available at this time but may be obtained from the authors upon reasonable request.

## References

[1] Lu W., Chen L. (2022). Computer vision for solid waste sorting: A critical review of academic research. Waste Manag..

[2] Vrancken C., Longhurst P.J., Wagland S.T. (2017). Critical review of real-time methods for solid waste characterisation: Informing material recovery and fuel production. Waste Manag..

[3] Kumar L.M., Pavan B., Kalyan P.V., Paul N.S., Prakruth R.S., Chinnu T. Design of an embedded based control system for efficient sorting of waste plastics using Near Infrared Spectroscopy. Proceedings of the 2014 IEEE International Conference on Electronics, Computing and Communication Technologies (CONECCT).

[4] Carrera B., Piñol V.L., Mata J.B., Kim K. (2022). A machine learning based classification models for plastic recycling using different wavelength range spectrums. J. Clean. Prod..

[5] Gundupalli S.P., Hait S., Thakur A. (2017). A review on automated sorting of source-separated municipal solid waste for recycling. Waste Manag..

[6] Verhoeven G. (2018). Multispectral and hyperspectral imaging. The Encyclopedia of Archaeological Sciences.

[7] Marshall I.J., Wallace B.C. (2019). Toward systematic review automation: A practical guide to using machine learning tools in research synthesis. Syst. Rev..

[8] Smidt E., Meissl K., Schwanninger M., Lechner P. (2008). Classification of waste materials using Fourier transform infrared spectroscopy and soft independent modeling of class analogy. Waste Manag..

[9] Erickson Z., Luskey N., Chernova S., Kemp C.C. (2019). Classification of household materials via spectroscopy. IEEE Robot. Autom. Lett..

[10] Rahman M.O., Hannan M.A., Scavino E., Hussain A., Basri H. (2009). An efficient paper grade identification method for automatic recyclable waste paper sorting. Eur. J. Sci. Res..

[11] Rani M., Marchesi C., Federici S., Rovelli G., Alessandri I., Vassalini I., Ducoli S., Borgese L., Zacco A., Bilo F. (2019). Miniaturized near-infrared (MicroNIR) spectrometer in plastic waste sorting. Materials.

[12] Kramer K., Ebel S. (2000). Application of NIR reflectance spectroscopy for the identification of pharmaceutical excipients. Anal. Chim. Acta.

[13] Ropodi A.I., Panagou E.Z., Nychas G.J. (2017). Multispectral imaging (MSI): A promising method for the detection of minced beef adulteration with horsemeat. Food Control.

[14] De Oliveira Penido C.A., Pacheco M.T., Lednev I.K., Silveira L. (2016). Raman spectroscopy in forensic analysis: Identification of cocaine and other illegal drugs of abuse. J. Raman Spectrosc..

[15] Wang X., Jiang S., Hu W., Ye S., Wang T., Wu F., Yang L., Li X., Zhang G., Chen X. (2022). Quantitatively determining surface-adsorbate properties from vibrational spectroscopy with interpretable machine learning. J. Am. Chem. Soc..

[16] Pudełko A., Chodak M., Roemer J., Uhl T. (2020). Application of FT-NIR spectroscopy and NIR hyperspectral imaging to predict nitrogen and organic carbon contents in mine soils. Measurement.

[17] Roberts J., Power A., Chapman J., Chandra S., Cozzolino D. (2018). A short update on the advantages, applications, and limitations of hyperspectral and Chemical Imaging in food authentication. Appl. Sci..

[18] Wu X., Li J., Yao L., Xu Z. (2020). Auto-sorting commonly recovered plastics from waste household appliances and electronics using near-infrared spectroscopy. J. Clean. Prod..

[19] Masoumi H., Safavi S.M., Khani Z. (2012). Identification and classification of plastic resins using near infrared reflectance. Int. J. Mech. Ind. Eng..

[20] Huang J., Pretz T., Bian Z. Intelligent solid waste processing using optical sensor based sorting technology. Proceedings of the 2010 3rd International Congress on Image and Signal Processing.

[21] Kulko R.D., Pletl A., Hanus A., Elser B. (2023). Detection of Plastic Granules and Their Mixtures. Sensors.

[22] Farcomeni A., Serranti S., Bonifazi G. (2008). Non-parametric analysis of infrared spectra for recognition of glass and glass ceramic fragments in recycling plants. Waste Manag..

[23] McWhirt A.L., Weindorf D.C., Chakraborty S., Li B. (2012). Visible near infrared diffuse reflectance spectroscopy (VisNIR DRS) for rapid measurement of organic matter in compost. Waste Manag. Res..

[24] Simola E.T., De Iacovo A., Frigerio J., Ballabio A., Fabbri A., Isella G., Colace L. (2019). Voltage-tunable dual-band Ge/Si photodetector operating in VIS and NIR spectral range. Opt. Express.

[25] Rozenstein O., Puckrin E., Adamowski J. (2017). Development of a new approach based on midwave infrared spectroscopy for post-consumer black plastic waste sorting in the recycling industry. Waste Manag..

[26] Jung Y. (2018). Multiple predicting K-fold cross-validation for model selection. J. Nonparametric Stat..

[27] Smith L.I. (2002). A Tutorial on Principal Components Analysis.

[28] Singh A., Thakur N., Sharma A. A review of supervised machine learning algorithms. Proceedings of the 2016 3rd International Conference on Computing for Sustainable Global Development (INDIACom).

[29] Heydarian M., Doyle T.E., Samavi R. (2022). MLCM: Multi-label confusion matrix. IEEE Access.

[30] Singh A., Pandey B. An euclidean distance based KNN computational method for assessing degree of liver damage. Proceedings of the 2016 International Conference on Inventive Computation Technologies (ICICT).

[31] Hong C.S., Oh T.G. (2021). TPR-TNR plot for confusion matrix. Commun. Stat. Appl. Methods.

